# Safety and efficacy of NOAC vs. VKA in patients treated by PCI: a retrospective study of the FRANCE PCI registry

**DOI:** 10.3389/fcvm.2023.1320001

**Published:** 2024-01-16

**Authors:** Eric Durand, Thibault Verrez, Andre Gillibert, Thomas Levesque, Thomas Barbe, René Koning, Pascal Motreff, Hélène Eltchaninoff, Jean-Philippe Collet, Gregoire Rangé

**Affiliations:** ^1^ Department of Cardiology, Normandie Univ, UNIROUEN, U1096, CHU Rouen, Rouen, France; ^2^ Department of Biostatistics, Normandie Univ, CHU Rouen, Rouen, France; ^3^ Department of Cardiology, Clinique Saint Hilaire, Rouen, France; ^4^ Department of Cardiology, Clermont Ferrand University Hospital, Clermont-Ferrand, France; ^5^ Sorbonne Université, ACTION Group, INSERM UMRS 1166, Hôpital Pitié-Salpêtrière (AP-HP), Institut de Cardiologie, Paris, France; ^6^ Department of Cardiology, Hôpital de Chartres, Chartres, France

**Keywords:** PCI, VKA, NOAC, bleeding, atrial fibrillation

## Abstract

**Introduction:**

Dual antithrombotic therapy (DAT) combining oral anticoagulation (OAC), preferentially Non-vitamin K antagonist OAC (NOAC) and single antiplatelet therapy (SAPT) for a period of 6–12 months is recommended after percutaneous coronary intervention (PCI) in patients with an indication for OAC.

**Objective:**

To compare outcomes between vitamin K antagonist (VKA) and NOAC-treated patients in the nation-wide France PCI registry.

**Methods:**

All consecutive patients from the France PCI registry treated by PCI and discharged with OAC between 2014 and 2020 were included and followed one-year. Major bleeding was defined as Bleeding Academic Research Consortium (BARC) classification ≥3 and major adverse cardiac events (MACE) as the composite of all-cause mortality, myocardial infarction (MI), and ischemic stroke. A propensity-score analysis was used.

**Results:**

Of the 7,277 eligible participants, 2,432 (33.4%) were discharged on VKA and 4,845 (66.6%) on NOAC. After propensity-score adjustment, one-year major bleeding was less frequent in NOAC vs. VKA-treated participants [3.1% vs. 5.2%, −2.1% (−3.6% to −0.6%), *p* = 0.005 as well as the rate of MACE [9.2% vs. 11.9%, −2.7% (−5.0% to −0.4%), *p* = 0.02]. One-year mortality was also significantly decreased in NOAC vs. VKA-treated participants [7.4% vs. 9.9%, −2.6% (−4.7% to −0.5%), *p* = 0.02]. The area under ROC curves of the anticoagulant treatment propensity score was estimated at 0.93, suggesting potential indication bias

**Conclusions:**

NOAC seems to have a better efficacy and safety profile than VKA. However, potential indication bias were found.

## Introduction

Patients with acute coronary syndrome (ACS) or who undergo percutaneous coronary intervention (PCI) require dual antiplatelet therapy (DAPT) with aspirin and a P2Y12 inhibitor to prevent myocardial infarction (MI) and stent thrombosis (1). The association of atrial fibrillation (AF) and coronary artery disease (CAD) is common and require the combination of oral anticoagulant (OAC) therapy to prevent ischaemic stroke and systemic embolism (2) with DAPT (3).

The AUGUSTUS trial (a two-by-two factorial, randomized, controlled clinical trial) demonstrated less bleeding in apixaban vs. VKA-treated AF patients with recent ACS or PCI [10.5% vs. 14.7%, hazard ratio, 0.69; 95% confidence interval (CI): 0.58–0.81; *p* < 0.001] on a background of concomitant P2Y_12_ inhibitor therapy for 6 months (4). There was also more bleeding events in aspirin vs. placebo-treated participants (16.1% vs. 9.0%; hazard ratio, 1.89; 95% CI: 1.59–2.24; *p* < 0.001) (4). Other trials comparing dual antithrombotic therapy (DAT), a combination of single antiplatelet therapy (SAPT) with a Non-vitamin K oral anticoagulant (NOAC), vs. triple antithrombotic therapy (TAT), a combination of VKA and DAPT in a similar setting, demonstrated a lower bleeding rate with DAT. However, none of these trials were designed to assess whether this was due to the use of NOAC or to aspirin cessation (5–7).

Our study aimed to evaluate the real-world setting in the nation-wide prospective FRANCE-PCI registry. Our objectives were to describe the use of VKA vs. NOAC over time and the one-year clinical outcome among participants who underwent PCI.

## Methods

### Study population

France PCI is a fully electronic, daily updated, high-quality, and low-cost national multicenter observational registry that includes consecutive patients undergoing coronary angiogram and/or PCI in 47 French centers (8, 9). France PCI is registered at clinicaltrials.org (NCT02778724) and all participants are informed of data collection and of the aims of the survey. Antithrombotic regimens were collected before PCI and after PCI at discharge. Participants who underwent PCI between January 2014 and December 2020 with an indication for long-term OAC at discharge were eligible for inclusion. Those without an indication for OAC or who had an ischemic or a bleeding event before discharge were excluded.

### Data collection and outcomes

More than 150 epidemiological, clinical and procedural variables are collected as well as 1-year follow-up of all patients who have undergone PCI. These data are extracted from reporting software and monitored to ensure exhaustivity and optimal quality control. Data monitoring, reporting and extraction are supervised by the coordinating clinical research associate and a national medical coordinator.

Participants follow-up is the responsibility of the local on-site research technician at each participating centers. Major adverse events (MACE) defined as the composite of death, stent thrombosis (ARC-2 definition), myocardial infarction (ESC definition), unplanned coronary revascularization, major bleeding (BARC ≥ 3 definition) (10) and stroke were assessed at one-year. The primary safety endpoint was major bleeding (BARC ≥ 3). The primary efficacy endpoint was a composite of all-cause mortality, MI, and ischemic stroke. Individual components of the primary efficacy endpoints as well as the net clinical benefit were also evaluated.

### Statistical analysis

A propensity score weighting, based on overlap weights and binary logistic regression explaining the exposure (NOAC vs. VKA or TAT vs. DAT) by adjustment variables was used (10). No survival model was required and endpoints were analyzed as binary variables given the exhaustive follow-up. Each treatment choice (anticoagulant and antiplatelet) was analyzed in a different propensity score analysis, but the other treatment was used as an adjustment variable. Other adjustment variables, listed in Table 1, were chosen *a priori*, as known prognostic factors or potential indication variables, and included in the propensity score. The year of admission (coded as a categorical variable from 2014 to 2020) was used as an adjustment variable in propensity scores analyses. Heteroscedasticity consistent of type 3 (HC3) sandwich estimator was used for propensity score analysis in a general linear model weighted by overlap weights of the propensity scored, explaining the binary endpoint by the binary exposure; multiple imputation with Rubin's rule for pooling variances was used to handle missing data.

**Table 1:** Adjustment variables included in the propensity score.

Variables	Measurements
Indication for PCI	STEMI, NSTEMI, CCS
Emergency	Yes/no
Patient's age at admission	<65, 65–70, 70–75, 75–85, 80–85, ≥85 years
Sex	Male/female
Number of stents	0, 1, 2, ≥3
Syntax score	quintiles coded as nominal categorical variable
Body mass index at admission	(<18.5, 18.5–25, 25–30, ≥30
History of myocardial infarction	Yes/no
History of ischemic stroke or transient ischemic attack	Yes/no
Hypertension	Yes/no
Renal failure	no failure, GFR > 50 ml/min, GFR 30–50, GFR < 30 without dialysis, dialysis
Diabetes	no, type I, type II
Tobacco use	never, former, current
LVEF before PCI	(<30, 30–40, 41–50, >50%
Anticoagulant use before admission	none, NOAC, VKA, Other
Antiplatelet use before admission	none, aspirin alone, other antiplatelet drug alone, aspirin + other antiplatelet drug)

To account for the potential indication bias, an ecological sensitivity analysis was performed. All patients admitted on the same year were considered as equally exposed to the treatment (NOAC vs. VKA). This analysis was performed using a general linear model explaining the 1-year endpoint (binary variable) by the exposition rate to NOAC on the admission year and by the same adjustment variables as the propensity score analysis, except the admission year; the usual variance estimator was used. The strength of the indication bias was assessed by the area under the Receiver Operating Characteristic (ROC) curve of the propensity score for the exposure.

## Results

### Number of participants

**Figure 1:**
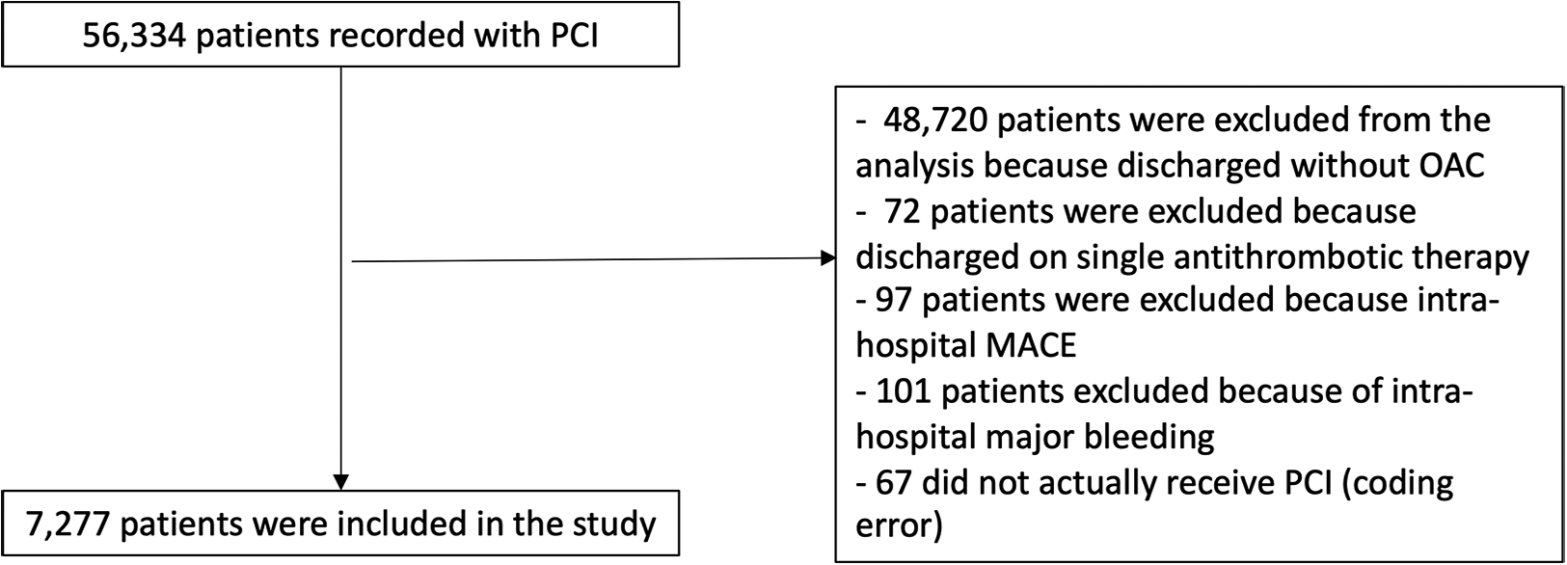
Study flowchart.

**Figure 2:**
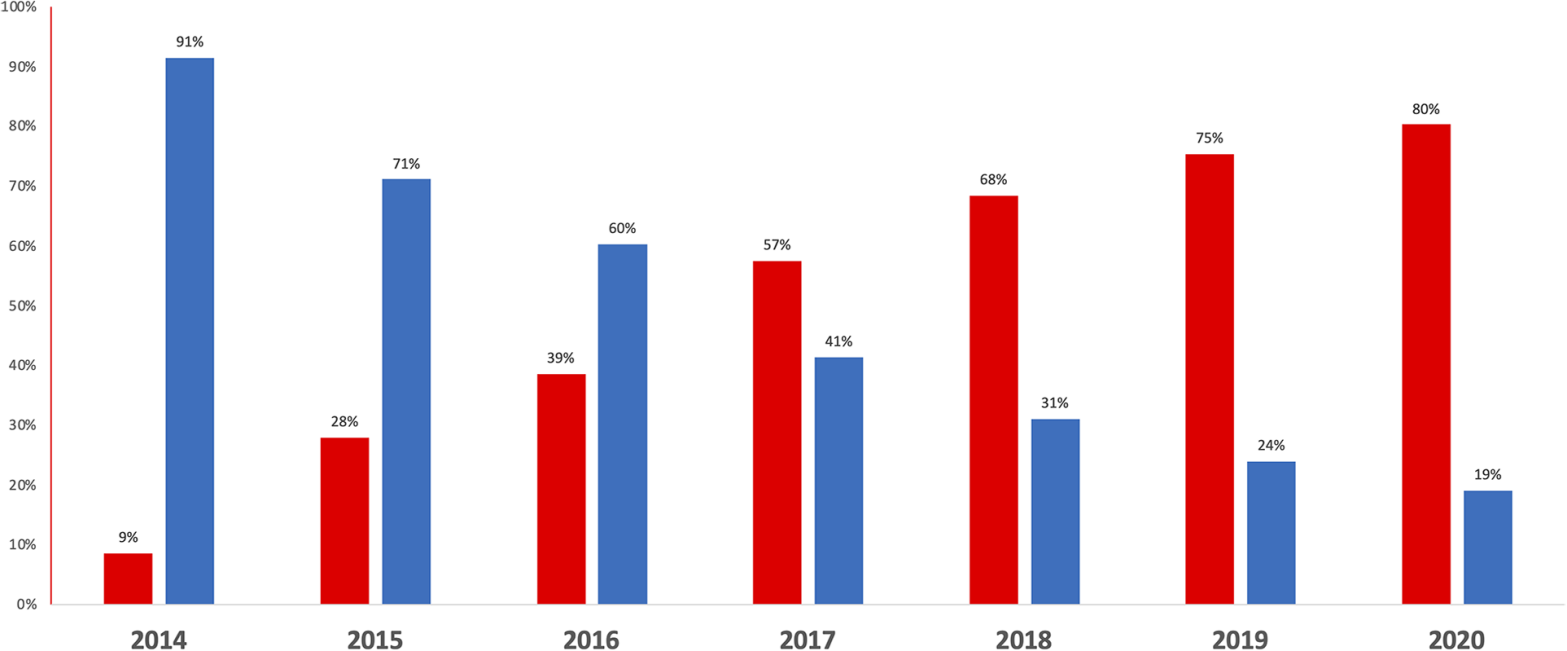
Proportion of patients prescribed long-term VKA (blue) and NOAC (red) at hospital discharge after PCI, between 2014 and 2020.

Between 2014 and 2020, 56,334 participants of the France-PCI registry were enrolled of whom 7,277 were event-free at discharge and exposed either to VKA or NOACs (Figure 1). The proportion of participants treated by NOAC vs. VKA increased dramatically during the study period (Figure 2). In 2017, the proportion of participants discharged on NOAC became superior to VKA (57% vs. 41%). The number of included participants also increased exponentially from 327 in 2014 to 2,528 in 2020 (Supplementary Table S1).

### Baseline characteristics

Baseline characteristics of the studied population according to VKA (*n* = 2,432) vs. NOAC (*n* = 4,845) regimen are shown in Table 2. VKA-treated participants were more comorbid than NOAC-treated participants with slightly higher CHA_2_DS_2_-VASc score and a more frequent exposure to antiplatelet therapy before admission. The indications of PCI were similar between the two groups but the femoral approach was more frequent in VKA vs. NOAC participants and the syntax score and the number of treated vessels was also significantly higher. The proportion of patients receiving a DAT at discharge was significantly higher in the NOAC group. The proportion of patients treated by TAT vs. DAT at hospital discharge slightly decreased over the study period, from 286/327 (87.5%) in 2014 to 1,914/2,528 (75.7%) in 2020.

**Table 2:** Baseline characteristics of patients treated with VKA and NOAC.

	VKA *N* = 2,432	NOAC *N* = 4,845	*p*-value
Demographics			
Age, years, *n* (%)			0.0007
<65	392 (16.1%)	704 (14.5%)	
65–69	290 (11.9%)	650 (13.4%)	
70–74	345 (14.2%)	844 (17.4%)	
75–79	466 (19.2%)	834 (17.2%)	
80–84	533 (21.9%)	986 (20.4%)	
≥85	406 (16.7%)	827 (17.1%)	
Female	570 (23.4%)	1,139 (23.5%)	0.97
BMI (kg/m^2^), *n*/*N* (%)			0.38
<18.5	27/2,404 (1.1%)	48/4,788 (1%)	
18.5–25	648/2,404 (27%)	1,380/4,788 (28.8%)	
25–30	1,006/2,404 (41.8%)	1,976/4,788 (41.3%)	
≥30	723/2,404 (30.1%)	1,384/4,788 (28.9%)	
Medical history			
Hypertension, *n*/*N* (%)	1,683/2,424 (69.4%)	3,285/4,834 (68%)	0.21
Dyslipidemia, *n*/*N* (%)	1,287/2,376 (54.2%)	2,398/4,745 (50.5%)	0.004
Diabetes, *n*/*N* (%)			<0.0001
Type I diabetes	239/2,424 (9.9%)	334/4,831 (6.9%)	
Type II	588/2,424 (24.3%)	1,105/4,831 (22.9%)	
Smoking, *n*/*N* (%)			0.34
Former smoker	675/2,423 (27.9%)	1,422/4,828 (29.5%)	
Active smoker	271/2,423 (11.2%)	514/4,828 (10.6%)	
Myocardial infarction, *n*/*N* (%)	423/2,421 (17.5%)	677/4,828 (14%)	0.0001
CABG, *n*/*N* (%)	285/2,429 (11.7%)	362/4,843 (7.5%)	<0.0001
PCI, *n*/*N* (%)	731/2,427 (30.1%)	1,424/4,839 (29.4%)	0.56
CHA_2_DS_2_-VASc, mean ± SD	4.03 ± 1.33	3.91 ± 1.31	0.0003
Stroke, *n*/*N* (%)	161/2,430 (6.6%)	315/4,838 (6.5%)	0.89
Peripheral artery disease, *n*/*N* (%)	365/2,419 (15.1%)	600/4,820 (12.4%)	0.002
Chronic kidney diease, *n*/*N* (%)			<0.0001
GFR > 50 ml/min	1,898/2,418 (78.5%)	4,185/4,796 (87.3%)	
GFR 30–50 ml/min	301/2,418 (12.4%)	541/4,796 (11.3%)	
GFR < 30 ml/min	120/2,418 (5%)	63/4,796 (1.3%)	
Dialysis	99/2,418 (4.1%)	7/4,796 (0.1%)	
OAC regimen before admission, *n*/*N* (%)			<0.0001
NOAC	44/2,412 (1.8%)	2,556/4,830 (52.9%)	
VKA	1,336/2,412 (55.4%)	218/4,830 (4.5%)	
Other	3/2,412 (0.1%)	7/4,830 (0.1%)	
None	1,029/2,412 (42.7%)	2,049/4,830 (42.4%)	
Antiplatelet therapy before admission, *n*/*N* %()			<0.0001
Aspirin	287/2,415 (11.9%)	720/4,835 (14.9%)	
Other antiplatelet drug	238/2,415 (9.9%)	500/4,835 (10.3%)	
Aspirin + Other antiplatelet drug	1,680/2,415 (69.6%)	3,018/4,835 (62.4%)	
None	210/2,415 (8.7%)	597/4,835 (12.3%)	
Indication for PCI, *n*/*N* (%)			0.38
Non-STEMI	826/2,431 (34%)	1,622/4,845 (33.5%)	
STEMI	200/2,431 (8.2%)	446/4,845 (9.2%)	
Chronic coronary syndrome	1,405/2,431 (57.8%)	2,777/4,845 (57.3%)	
PCI performed in emergency, *n*/*N* (%)	1,267/2,431 (52.1%)	2,606/4,845 (53.8%)	0.19
LVEF before PCI, *n*/*N* (%)			<0.0001
<30, *n* (%)	231/2,063 (11.2%)	355/3,996 (8.9%)	
30–40, *n* (%)	345/2,063 (16.7%)	530/3,996 (13.3%)	
41–50, *n* (%)	367/2,063 (17.8%)	665/3,996 (16.6%)	
>50, *n* (%)	1,120/2,063 (54.3%)	2,446/3,996 (61.2%)	
Unknown, *n* (%)	369/2,432 (15.2%)	849/4,845 (17.5%)	
Procedure			
Angiographic results, *n*/*N* (%)			
Monotroncular	817/2,419 (33.8%)	1,658/4,817 (34.4%)	0.093
Bitroncular	821/2,419 (33.9%)	1,664/4,817 (34.5%)	
Tritroncular	734/2,419 (30.3%)	1,365/4,817 (28.3%)	
Left main alone	18/2,419 (0.7%)	36/4,817 (0.7%)	
All lesions <50%	29/2,419 (1.2%)	94/4,817 (2%)	
Left main (alone or not)	210/2,430 (8.6%)	373/4,845 (7.7%)	0.18
Syntax score, *n*/*N* (%)			0.001
[0,5]	495/2,249 (22%)	1,085/4,306 (25.2%)	
[6,9]	476/2,249 (21.2%)	986/4,306 (22.9%)	
[10,17]	664/2,249 (29.5%)	1,192/4,306 (27.7%)	
[18,110]	614/2,249 (27.3%)	1,043/4,306 (24.2%)	
Unknown	183/2,432 (7.5%)	539/4,845 (11.1%)	
Approach, *n*/*N* (%)			<0.0001
Femoral	308/2,430 (12.7%)	404/4,844 (8.3%)	
Radial	2,097/2,430 (86.3%)	4,412/4,844 (91.1%)	
Other	25/2,430 (1%)	28/4,844 (0.6%)	
Anticoagulation during PCI, *n*/*N* (%)			<0.0001
UFH	2,006/2,419 (82.9%)	4,064/4,831 (84.1%)	
LMWH	92/2,419 (3.8%)	344/4,831 (7.1%)	
Other	9/2,419 (0.4%)	15/4,831 (0.3%)	
None	312/2,419 (12.9%)	408/4,831 (8.4%)	
Number of treated vessels, *n* (%)			0.033
1	1,408 (57.9%)	2,870 (59.2%)	
2	649 (26.7%)	1,337 (27.6%)	
≥3	375 (15.4%)	638 (13.2%)	
Number of stents, *n* (%)			0.088
0	151 (6.2%)	289 (6%)	
1	1,305 (53.7%)	2,664 (55%)	
2	617 (25.4%)	1,278 (26.4%)	
≥3	359 (14.8%)	614 (12.7%)	
Fluoroscopy time (min), mean ± SD	12.92 ± 10.89	12.56 ± 9.93	0.16
Discharge medication			
VKA, *n* (%)	2,432 (100%)	0	
NOAC, *n* (%)	0	4,845 (100%)	
Apixaban		2,726 (56.3%)	
Rivaroxaban		1,847 (38.1%)	
Dabigatran		272 (5.6%)	
Antiplatelet therapy, *n* (%)			<0.0001
Clopidogrel alone	254 (10.4%)	1,248 (25.8%)	
Aspirin alone	73 (3%)	77 (1.6%)	
Ticagrelor alone	15 (0.6%)	33 (0.7%)	
Clopidogrel + Aspirin	2,005 (82.4%)	3,405 (70.3%)	
Ticagrelor + Aspirin	75 (3.1%)	76 (1.6%)	
Prasugrel + Aspirin	10 (0.4%)	6 (0.1%)	
Antiplatelet treatment, *n* (%)			<0.0001
DAT	342 (14.1%)	1,358 (28%)	
TAT ≤ 1 month	237 (9.7%)	377 (7.8%)	
TAT > 1 month	1,612 (66.3%)	2,777 (57.3%)	
TAT with unknown duration	241 (9.9%)	333 (6.9%)	

BMI, body mass index; CABG, coronary artery bypass graft; PCI, percutaneous coronary intervention; GFR, glomerular filtration rate; NOAC, non-vitamin K antagonist oral anticoagulant; VKA, vitamin K antagonist; STEMI, ST elevation myocardial infarction; LVEF, left ventricular ejection fraction; UFH, unfractionated heparin; LMWH, low molecular weight heparin; DAT, dual antithrombotic therapy; TAT, triple antithrombotic therapy; SD, standard deviation.

### Outcomes according to VKA vs. NOAC

The area under ROC curves of the anticoagulant treatment propensity score was estimated at 0.930 (or 0.896 if the admission year is removed from the propensity score), suggesting that the potential indication bias could be major. The fraction of missing information, according to multiple imputation was estimated at 0.38% for the primary outcome.

**Figure 3:**
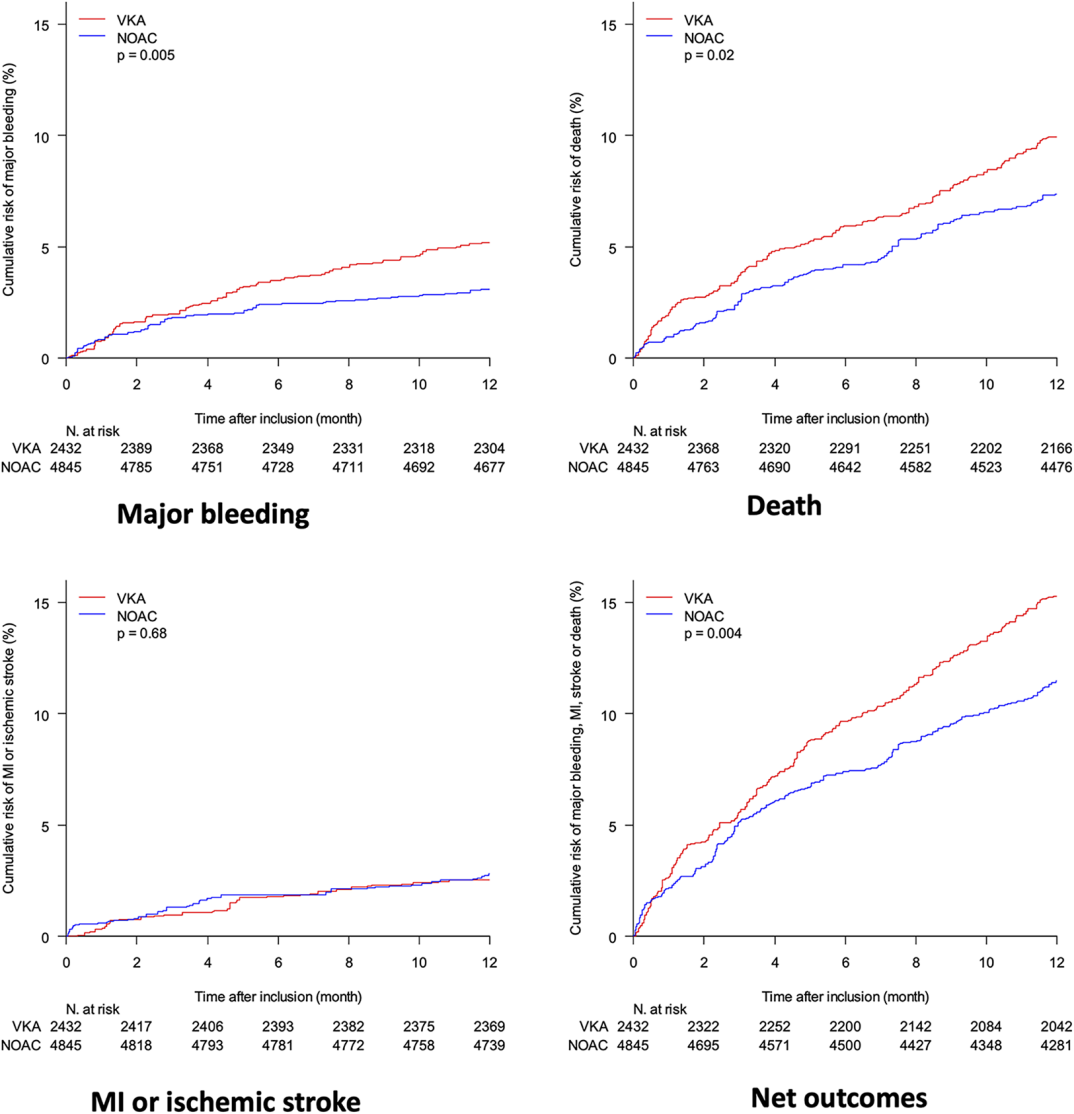
Comparison of propensity-score weighted cumulative risk of major bleeding, death, myocardial infraction (MI) and stroke and net outcomes (including death, MI, stroke and major bleeding) between patients on VKA (red) and NOAC (blue); adjustment variables are the same as in the primary analysis, including year of admission.

The 12-months incidence of major bleeding was significantly lower in the NOAC group, a difference that was sustained after propensity-score adjustment (Table 3 and Figure 3). The incidence of death, MI and ischemic stroke was significantly lower in the NOAC group, a difference driven by a significant reduction of death halved (−4.5% to −2.6%) by propensity score adjustments. The net clinical benefit was similar between the two groups.

**Table 3:** Minimally adjusted and fully adjusted 12-months clinical outcomes of patients prescribed long-term NOAC *versus* VKA at hospital discharge of a PCI, with propensity score weighting and multiple imputation.

	Minimally adjusted analysis (propensity score)^a^					Fully adjusted analysis (propensity score)^b^				
	VKA (*N* = 2,432)	NOAC (*N* = 4,845)	Absolute risk reduction	95% CI	*p*-value	VKA (*N* = 2,432)	NOAC (*N* = 4,845)	Absolute risk reduction	95% CI	*p*-value
Bleeding events										
Major bleeding >BARC 3^c^	5.6%	3.3%	−2.3%	(−3.4% to −1.1%)	<0.0001	5.2%	3.1%	−2.1%	(−3.6% to −0.6%)	0.005
Ischemic events										
Death, MI, ischemic stroke^d^	13.4%	8.5%	−4.9%	(−6.7% to −3.2%)	<0.0001	11.9%	9.2%	−2.7%	(−5.0% to −0.4%)	0.02
Death	11.5%	7.0%	−4.5%	(−6.1% to −2.9%)	<0.0001	9.9%	7.4%	−2.6%	(−4.7% to −0.5%)	0.02
MI	1.8%	1.2%	−0.6%	(−1.3%–0.1%)	0.07	1.6%	1.8%	0.1%	(−0.9%–1.2%)	0.78
Ischemic stroke	0.9%	0.9%	0.0%	(−0.5%–0.5%)	0.91	0.9%	1.1%	0.1%	(−0.6%–0.9%)	0.71
Ischemic stroke, MI	2.7%	2.1%	−0.6%	(−1.5%–0.2%)	0.16	2.5%	2.8%	0.3%	(−1.0%–1.5%)	0.68
Stent thrombosis	0.3%	0.4%	0.1%	(−0.2%–0.4%)	0.69	0.4%	0.5%	0.2%	(−0.3%–0.6%)	0.53
Death, MI, ischemic stroke, stent thrombosis, unplanned PCI	16.6%	11.8%	−4.8%	(−6.8% to −2.9%)	<0.0001	14.8%	12.7%	−2.1%	(−4.7%–0.5%)	0.11
Bleeding and ischemic events										
Major bleeding, death, MI, stroke	16.9%	10.9%	−6.0%	(−7.9% to −4.1%)	<0.0001	15.3%	11.5%	−3.8%	(−6.3% to −1.2%)	0.004

MI, myocardial infarction; PCI, percutaneous coronary intervention.

^a^ Primary safety endpoint.

^b^ Primary efficacy endpoint.

^c^ Adjusted on year of admission.

^d^ Adjusted on year of admission, PCI indication, emergency/planned PCI, age, sex, number of stents, syntax score (quintiles), body mass index, history of myocardial infraction, history of stroke/transient ischemic attack, hypertension before admission, renal failure, diabetes, tobacco use, LVEF, anticoagulant treatment before admission, antiplatelet treatment before admission, antiplatelet treatment at discharge (TAT vs DAT).

Outcomes did not change according to the year of inclusion (Supplementary Figure S1). The fully adjusted ecological sensitivity analysis did not find a significant effect of the NOAC prescription rate on the 12-months risk of major bleeding (+0.1%, 95% CI: −2.3% to +2.5%, *p* = 0.94), composite endpoint of death, MI and ischemic stroke (+1.7%, 95% CI: −2.0% to +5.3%, *p* = 0.37), or any other endpoint (Supplementary Table S2). However, confidence intervals were wider than in the primary analysis suggesting some loss of statistical power.

### Outcomes according to DAT vs. TAT

Propensity score adjusted analyses of the effect of TAT vs. DAT are shown in Table 4. The area under ROC curve of the associated propensity score was 0.774. The composite endpoint of major bleeding, death, MI and stroke was lower in TAT vs. DAT (−3.0%, 95% CI: −4.9% to −1.1%, *p* = 0.002) in the minimally adjusted model, but this effect was attenuated and became non-significant after full adjustment (−2.0%, 95% CI: −4.1% to +0.1%, *p* = 0.07).

**Table 4:** Minimally adjusted and fully adjusted 12-months clinical outcomes of patients prescribed TAT vs. DAT at hospital discharge of a PCI, with propensity score weighting and multiple imputation.

	Minimally adjusted analysis (propensity score)^a^					Fully adjusted analysis (propensity score)^b^				
	DAT (*N* = 1,700)	TAT (*N* = 5,577)	Absolute risk reduction	95% CI	*p*-value	DAT (*N* = 1,700)	TAT (*N* = 5,577)	Absolute risk reduction	95% CI	*p*-value
Bleeding events										
Major bleeding > BARC 3	4.6%	3.8%	−0.8%	(−1.9%–0.3%)	0.16	4.4%	4.1%	−0.3%	(−1.6%–0.9%)	0.58
Ischemic events										
Death, MI, ischemic stroke	11.7%	9.6%	−2.2%	(−3.9% to −0.4%)	0.02	11.6%	10.1%	−1.5%	(−3.4%–0.4%)	0.12
Death	9.8%	8.0%	−1.8%	(−3.4% to −0.1%)	0.03	9.7%	8.7%	−1.0%	(−2.8%–0.8%)	0.26
MI	1.2%	1.5%	0.3%	(−0.3%–0.9%)	0.34	1.3%	1.4%	0.2%	(−0.5%–0.8%)	0.66
Ischemic stroke	1.1%	0.8%	−0.3%	(−0.9%–0.2%)	0.26	1.0%	0.9%	−0.2%	(−0.8%–0.4%)	0.58
Ischemic stroke, MI	2.4%	2.3%	0.0%	(−0.9%–0.8%)	0.92	2.3%	2.3%	0.0%	(−0.9%–0.9%)	0.95
Stent thrombosis	0.4%	0.3%	−0.1%	(−0.5%–0.2%)	0.41	0.4%	0.3%	−0.1%	(−0.5%–0.2%)	0.50
Death, MI, ischemic stroke, stent thrombosis, unplanned PCI	14.1%	12.9%	−1.2%	(−3.1%–0.7%)	0.23	13.9%	13.3%	−0.5%	(−2.6%–1.5%)	0.60
Bleeding and ischemic events										
Major bleeding, death, MI, stroke	15.1%	12.1%	−3.0%	(−4.9% to −1.1%)	0.002	14.9%	13.0%	−2.0%	(−4.1%–0.1%)	0.07

^a^ Adjusted on year of admission.

^b^ Adjusted on year of admission, PCI indication, emergency/planned PCI, age, sex, number of stents, syntax score (quintiles), body mass index, history of myocardial infraction, history of stroke/transient ischemic attack, hypertension before admission, renal failure, diabetes, tobacco use, LVEF, anticoagulant treatment before admission, antiplatelet treatment before admission, anticoagulant treatment at discharge (NOAC vs VKA).

In linear models adjusted on the same variables as the primary analysis with exposure defined as the actual treatment that the patient was prescribed, there was no significant statistical interaction on any outcome although statistical precision of the interaction term was low (Supplementary Table S3).

## Discussion

The main results of our study may be summarized as follow: (1) The proportion of patients treated with NOAC increased dramatically between 2014 and 2020; (2) Major bleeding and MACE were significantly lower in NOAC vs. VKA-treated participants. (3) Mortality was also lower, a difference not accounted by major bleeding reduction; (4) There was no statistical interaction with TAT and DAT regimen for all outcomes.

### NOAC vs. VKA

From 2014 to 2021, the European guidelines concerning the optimal antithrombotic regimen in patients with long-term OAC treated with PCI have evolved from a TAT including an OAC and DAPT to a DAT including an OAC (preferentially a NOAC) and SAPT (preferentially clopidogrel) for a period of 6–12 months according to the clinical context and bleeding risk profile of the patient. These recommendations are based on the results of randomized multicenter studies (WOEST, PIONEER AF-PCI, RE-DUAL PCI, and ENTRUST-AF PCI trials) demonstrating lower bleeding outcomes with DAT as compared to TAT without increase in the risk of ischemic events (5–7, 11).

Our results are in accordance with the AUGUSTUS trial showing a significant reduction of major bleeding in patients treated with apixaban as compared to those on VKA [hazard ratio (HR) 0.69, 95% CI: 0.58–0.81, *p* < 0.001] (4). Unlike our study, the incidence of MACE was not significantly different between the apixaban and VKA groups (HR, 0.93, 95% CI: 0.75–1.16, *p* = NS) (4). Other randomized trials have compared a DAT including a single antiplatelet therapy and a NOAC vs. TAT including a vitamin K antagonist (VKA) and a dual antiplatelet therapy in patients with AF who were undergoing PCI. Those studies showed a lower incidence of bleeding with DAT but these three trials were not designed to assess whether the lower incidence of bleeding was due to the use of the standard or reduced doses of NOAC or to the removal of aspirin therapy (5–7).

A meta-analysis of benefits and risks associated with the use of NOAC vs. VKA in patients treated by PCI has been therefore reported (12). The primary outcome (composite of major bleeding according to ISTH definition and clinically relevant bleeding requiring medical intervention) occurred less frequently in patients receiving NOAC. Combination strategies with NOACs were associated with reduced risk of major bleeding events across different combination strategies as compared to VKA, with the most significant risk reduction when NOAC + SAPT was associated with a 37% relative risk reduction of major bleeding events as compared to TAT with VKA + DAPT (RR, 0.63; 95% CI: 0.50–0.80) (12). The reduction of major bleeding risks was considered as a class effect of NOACs. Again, unlike our study, combination strategies of NOAC vs. VKA resulted in a comparable risk of MACE. In our study, the incidence of MI and stroke was similar in patients receiving NOAC or VKA. The significant reduction of MACE was only driven by a significant reduction of the incidence of death. A *post hoc* analysis excluding all patients who had a major bleeding found an absolute difference of 1-year death at −3.79% (−5.36% to −2.22%, *p* < 0.0001) for NOAC vs. VKA without adjustment; after propensity-score adjustment it was −1.95% (−3.99%–0.09%, *p* = 0.06) for NOAC vs. VKA, confirming the hypothesis that most of the death reduction is unrelated to bleeding events. Moreover, the strong attenuation of this effect after statistical adjustments and disappearance in the ecological sensitivity analysis, suggest that this effect may be partly or fully explained by residual confounding.

### Antiplatelet regimen

In our study, we did not find any significant effect of TAT vs. DAT. This result is not in accordance with the randomized studies and meta-analysis showing that DAT, particularly the association of a NOAC and a P2Y12 inhibitor, was associated with significantly less major or clinically relevant non-major bleeding compared to TAT (4–7, 11–13). There are several explanations for this result. First of all, only 23.4% of the studied population received a DAT between 2014 and 2020, leading to poor statistical precision. On the other hand, minimally adjusted analyses show that there was an indication bias with DAT given to patients with poor prognosis, so that the residual confounding bias may disadvantage DAT. Similarly, effects of TAT vs. DAT on the composite outcome of bleeding, death, MI or stroke, suggest that there were strong confounding factors, partly attenuated by adjustment.

Although for most of the duration of the study it was recommended to use a TAT by default for 1 month except in patients at high risk of bleeding, most patients receiving TAT had a prescription lasting more than 1 month. The latest guidelines recommend to use, as a default strategy, a DAT but were published in 2021.

### Strengths and limitations

The main strength is the large sample size and the use of a multi-center monitored prospective registry with high quality follow-up data at 12-months and real-life patients with usual care practice. Our study has several limitations. First, this analysis was retrospectively conducted. Second, the indication bias remained uncontrolled as suggested by an attenuation of effects after adjustment, with a risk of residual confounding due to unobserved confounders and measurement errors on observed confounders. Third, the ecological analysis was not affected by indication bias but had lower statistical power and may have differential selection or measurement bias due to evolution of the quality of the registry over time. Fourth, the antithrombotic regimen strategy was not collected at the time of PCI but at discharge with potential unmeasured confounders which may have interacted with the decision. Fifth, NOAC dose regimen and VKA monitoring were not collected. Sixth, the indication of anticoagulant therapy was not available in our database but the vast majority of patients had atrial fibrillation. Finally, events were not adjudicated and whether events occurred on-treatment could not be assessed.

## Conclusion

In the real-life setting, the use of NOAC vs. VKA after PCI was associated with less bleeding and less MACE at one-year follow-up. The benefit effect on MACE was mainly driven by a better survival. However, these effects should be interpreted with caution given that the indication of OAC remains a major bias with a risk of residual confounding due to unobserved confounders and measurement errors on observed confounders.

## Data Availability

The original contributions presented in the study are included in the article/**Supplementary Material**, further inquiries can be directed to the corresponding author.
