# Supplementary material for: Safety and efficacy of NOAC vs. VKA in patients treated by PCI: a retrospective study of the FRANCE PCI registry
Source: Front Cardiovasc Med. 2024 Jan 16;10:1320001. doi: 10.3389/fcvm.2023.1320001 (PMC10824844; doi:10.3389/fcvm.2023.1320001)
Supplement: Supplementary file 1 [file Table1.docx]

**France PCI Investigators**

Dr Louis Vialliard, CH Aurillac, [l.viallard@ch-aurillac.fr](mailto:l.viallard@ch-aurillac.fr)

Dr Matthieu Godin, Clinique Saint Hilaire, Rouen, [mgodin@clinique-sainthilaire.fr](mailto:mgodin@clinique-sainthilaire.fr)

Pr Farzin Beygui, CHU Caen, [beygui-f@chu-caen.fr](mailto:beygui-f@chu-caen.fr)

Dr Sofiéne Gafsi, CH Le Havre, [sofiene.gafsi@ch-havre.fr](mailto:sofiene.gafsi@ch-havre.fr)

Dr Pierre Francois Lesault, Hôpital estuaire Le Havre, [pflesault@gmail.com](mailto:pflesault@gmail.com)

Dr Gerard Dutheil, Clinique Bergougnian , Evreux, [gerard.dutheil01@orange.fr](mailto:gerard.dutheil01@orange.fr)

Dr Emmanuel Boiffard, CHD La Roche sur Yon, [emmanuel.boiffard@chd-vendee.fr](mailto:emmanuel.boiffard@chd-vendee.fr)

Dr Jean François Morelle, Hôpital Privé Saint Martin, Caen, [jeanfrancoismorelle@gmail.com](mailto:jeanfrancoismorelle@gmail.com)

Dr Antoine Py, Clinique cardiologie urgence, Amiens , [antoine_py@yahoo.fr](mailto:antoine_py@yahoo.fr)

Pr Jacques Monsegu GHM Grenoble, [jacquesmonsegu@gmail.com](mailto:jacquesmonsegu@gmail.com)

Pr Gilles Barrone- Rochette, CHU Grenoble, [GBarone@chu-grenoble.fr](mailto:GBarone@chu-grenoble.fr)

Dr Nicolas Amabile, IMM, Paris, [Nicolasamabile@yahoo.fr](mailto:Nicolasamabile@yahoo.fr)

Dr Sylvain Chanseaume, CH Montluçon, [s.chanseaume@ch-montlucon.fr](mailto:s.chanseaume@ch-montlucon.fr)

Dr Nicolas Durel, Pôle santé République, Clermont Ferrand, [dureln@yahoo.fr](mailto:dureln@yahoo.fr)

Dr Vincent Tixier, CH Vichy, [Vincent.Tixier@ch-vichy.fr](mailto:Vincent.Tixier@ch-vichy.fr)

Dr Stephane Rias, CH Chambery, [stephane.rias@ch-metropole-savoie.fr](mailto:stephane.rias@ch-metropole-savoie.fr)

Dr Ali Sharareh, Clinique Trélazé, Angers, [sha2.a2@orange.fr](mailto:sha2.a2@orange.fr)

Dr Benjamin Honton, Clinique Pasteur, Toulouse,  [bhonton@clinique-pasteur.com](mailto:bhonton@clinique-pasteur.com)
