# Supplementary material for: Safety and efficacy of NOAC vs. VKA in patients treated by PCI: a retrospective study of the FRANCE PCI registry
Source: Front Cardiovasc Med. 2024 Jan 16;10:1320001. doi: 10.3389/fcvm.2023.1320001 (PMC10824844; doi:10.3389/fcvm.2023.1320001)
Supplement: Supplementary file 2 [file Table2.docx]

**Supplementary Table 1.** Anticoagulant and antiplatelet prescription rates on discharge of PCI by year of admission

|  | **DAT+NOAC**  **n / N (%)** | **DAT+VKA**  **n / N (%)** | **TAT+NOAC**  **n / N (%)** | **TAT+VKA**  **n / N (%)** | **TAT overall**  **n / N (%)** | **NOAC overall**  **n / N (%)** |
| --- | --- | --- | --- | --- | --- | --- |
| Year |  |  |  |  |  |  |
| 2014 | 7 / 327 (2.1%) | 34 / 327 (10.4%) | 21 / 327 (6.4%) | 265 / 327 (81%) | 286 / 327 (87.5%) | 28 / 327 (8.6%) |
| 2015 | 24 / 397 (6.0%) | 25 / 397 (6.3%) | 88 / 397 (22.2%) | 260 / 397 (65.5%) | 348 / 397 (87.7%) | 112 / 397 (28.2%) |
| 2016 | 27 / 553 (4.9%) | 24 / 553 (4.3%) | 189 / 553 (34.2%) | 313 / 553 (56.6%) | 502 / 553 (90.8%) | 216 / 553 (39.1%) |
| 2017 | 108 / 688 (15.7%) | 23 / 688 (3.3%) | 292 / 688 (42.4%) | 265 / 688 (38.5%) | 557 / 688 (81%) | 400 / 688 (58.1%) |
| 2018 | 208 / 968 (21.5%) | 45 / 968 (4.6%) | 458 / 968 (47.3%) | 257 / 968 (26.5%) | 715 / 968 (73.9%) | 666 / 968 (68.8%) |
| 2019 | 455 / 1816 (25.1%) | 106 / 1816 (5.8%) | 923 / 1816 (50.8%) | 332 / 1816 (18.3%) | 1255 / 1816 (69.1%) | 1378 / 1816 (75.9%) |
| 2020 | 529 / 2528 (20.9%) | 85 / 2528 (3.4%) | 1516 / 2528 (60.0%) | 398 / 2528 (15.7%) | 1914 / 2528 (75.7%) | 2045 / 2528 (80.9%) |
| Overall | 1358 / 7277 (18.7%) | 342 / 7277 (4.7%) | 3487 / 7277 (47.9%) | 2090 / 7277 (28.7%) | 5577 / 7277 (76.6%) | 4845 / 7277 (66.6%) |

**Supplementary Table 2.** Ecological sensitivity analysis of 12-months outcomes were all patients admitted on the same year are considered as equally exposed to the anticoagulant treatment according to the proportion of patients who received this prescription on this year (i.e. exposition is averaged), in general linear models of which the slopes can be interpreted as the effect of NOAC vs VKA (difference between 0% and 100% of prescription of NOAC)

|  | **Uadjusted analysis (linear model)** | | | | | **Fully adjusted analysis (linear model)†** | | | | |
| --- | --- | --- | --- | --- | --- | --- | --- | --- | --- | --- |
|  | **VKA**  **(N=2432)** | **NOAC**  **(N=4845)** | **Absolute risk reduction** | **95%**  **CI** | **p-value** | **VKA**  **(N=2432)** | **NOAC**  **(N=4845)** | **Absolute risk reduction** | **95%**  **CI** | **p-value** |
| **Bleeding events** | | | | | | | | | | |
| Major bleeding > BARC 3 | 4.2% | 4.0% | -0.1% | (-2.5 to 2.2%) | 0.91 | 4.0% | 4.1% | 0.1% | (-2.3 to 2.5%) | 0.94 |
| **Ischemic events** | | | | | | | | | | |
| Death, MI, ischemic stroke | 9.2% | 10.9% | 1.8% | (-1.8 to 5.4%) | 0.32 | 9.2% | 10.9% | 1.7% | (-2.0 to 5.3%) | 0.37 |
| Death | 8.1% | 9.0% | 0.9% | (-2.4 to 4.2%) | 0.60 | 8.3% | 8.9% | 0.6% | (-2.8 to 4.0%) | 0.72 |
| MI | 0.9% | 1.7% | 0.8% | (-0.6 to 2.2%) | 0.28 | 0.7% | 1.8% | 1.2% | (-0.3 to 2.6%) | 0.12 |
| Ischemic stroke | 1.0% | 0.8% | -0.2% | (-1.3 to 1.0%) | 0.79 | 1.0% | 0.9% | -0.1% | (-1.3 to 1.0%) | 0.83 |
| Ischemic stroke, MI | 2.0% | 2.5% | 0.6% | (-1.2 to 2.3%) | 0.54 | 1.7% | 2.6% | 1.0% | (-0.9 to 2.8%) | 0.31 |
| Death, MI, ischemic stroke, stent thrombosis, unplanned PCI | 14.6% | 12.9% | -1.6% | (-5.6 to 2.4%) | 0.43 | 14.6% | 12.9% | -1.6% | (-5.8 to 2.5%) | 0.43 |
| **Bleeding and ischemic events** | | | | | | | | | | |
| Major bleeding, death, MI, stroke | 12.4% | 13.4% | 1.0% | (-2.9 to 5.0%) | 0.62 | 12.5% | 13.4% | 0.9% | (-3.1 to 5.0%) | 0.65 |

† adjusted on PCI indication, emergency/planned PCI, age, sex, number of stents, syntax score (quintiles), body mass index, history of myocardial infraction, history of stroke/transient ischemic attack, hypertension before admission, renal failure, diabetes, tobacco use, LVEF, anticoagulant treatment before admission, antiplatelet treatment before admission, antiplatelet treatment at discharge (TAT vs DAT)

**Supplementary Table 3.** Analysis of the interaction between anticoagulant and antiplatelet regimens, expressed as absolute risk differences in a general linear model explaining the outcome by the TAT, VKA, the interaction between the two, and all adjustment variables of the primary analysis

|  | **Fully adjusted linear model** | | | | |
| --- | --- | --- | --- | --- | --- |
|  | **Reference**  **(DAT + NOAC)**  **(N=1358)** | **DAT + VKA *vs* ref**  **(VKA effect alone)**  **(N=342)** | **TAT + NOAC *vs* ref**  **(TAT effect alone)**  **(N=3487)** | **TAT + VKA *vs* ref**  **(combined effects)**  **(N=2090)** | **Interaction***  **(N=7277)** |
| Major bleeding > BARC 3 | +0% | -0.35% (-2.94 to 2.25) p=0.79 | -0.98% (-2.30 to 0.33%)  p=0.14 | 1.29% (-0.45 to 3.02%) p=0.15 | 2.62% (0.02 to 5.22%) p=0.05 |
| Death, MI, ischemic stroke | +0% | 1.15% (-2.77 to 5.06%) p=0.57 | -1.71% (-3.69 to 0.28%) p=0.09 | 1.05% (-1.57 to 3.67%) p=0.43 | 1.61% (-2.32 to 5.54%) p=0.42 |
| Death | +0% | 1.93% (-1.69 to 5.55%) p=0.30 | -1.00% (-2.83 to 0.83%) p=0.28 | 1.65% (-0.77 to 4.07%) p=0.18 | 0.72% (-2.91 to 4.35%) p=0.70 |
| MI† | +0% | -1.06% (-2.63 to 0.51%) p=0.18 | -0.11% (-0.9 to 0.69%) p=0.79 | -0.11% (-1.16 to 0.93%) p=0.83 | 1.05% (-0.52 to 2.62%) p=0.19 |
| Ischemic stroke† | +0% | -0.60% (-1.84 to 0.64%) p=0.34 | -0.25% (-0.88 to 0.37%) p=0.43 | -0.46% (-1.29 to 0.37%) p=0.28 | 0.40% (-0.84 to 1.64%) p=0.53 |
| Ischemic stroke, MI | +0% | -1.65% (-3.63 to 0.33%) p=0.10 | -0.38% (-1.38 to 0.62%) p=0.46 | -0.56% (-1.88 to 0.76%) p=0.41 | 1.47% (-0.52 to 3.46%) p=0.15 |
| Death, MI, ischemic stroke, stent thrombosis, unplanned PCI | +0% | 1.22% (-3.20 to 5.64%) p=0.59 | -0.51% (-2.75 to 1.72%) p=0.65 | 1.37% (-1.58 to 4.32%) p=0.36 | 0.67% (-3.77 to 5.10%) p=0.77 |
| Major bleeding, death, MI, stroke | +0% | 1.49% (-2.86 to 5.83%) p=0.50 | -2.37% (-4.57 to -0.17%) p=0.03 | 1.35% (-1.55 to 4.26%) p=0.36 | 2.24% (-2.12 to 6.60%) p=0.31 |

* the interaction term is equal to the combined effect minus the sum of the VKA effect alone and TAT effect alone; *i.e.* it is the synergistic effect of VKA and TAT.

ref: reference (i.e. DAT + NOAC)

† statistical validity is not guaranteed as fewer than 5 events were expected in the DAT+VKA group with the hypothesis of independence of the outcome with the group
