# Supplementary figures and images for: Safety and efficacy of NOAC vs. VKA in patients treated by PCI: a retrospective study of the FRANCE PCI registry
Source: Front Cardiovasc Med. 2024 Jan 16;10:1320001. doi: 10.3389/fcvm.2023.1320001 (PMC10824844; doi:10.3389/fcvm.2023.1320001)

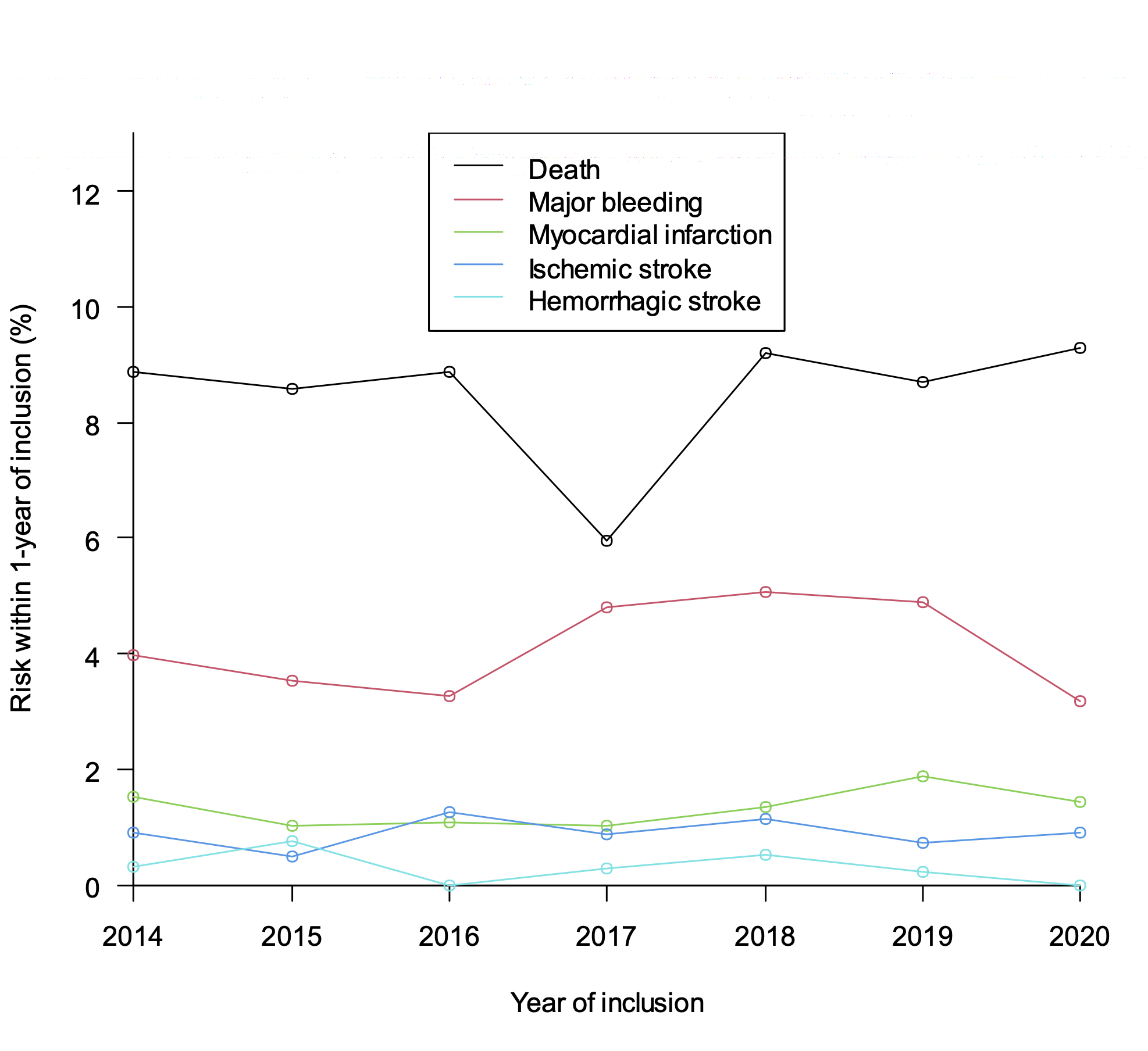

Supplement: Supplementary file 3 [file Image1.tiff]
